# FOXA1 expression and survival outcomes in triple-negative breast cancer: A retrospective cohort study

**DOI:** 10.1371/journal.pone.0362480

**Published:** 2026-11-13

**Authors:** Angela V. Fonseca-Benitez, David Diaz-Baez, Giulia Capella, Milena Rondón-Lagos, Isabella Castellano, Nelson Rangel

**Affiliations:** 1 Departamento de Nutrición y Bioquímica, Facultad de Ciencias, Pontificia Universidad Javeriana, Bogotá, Colombia; 2 INQA, Facultad de Ciencias Universidad El Bosque, Bogotá, Colombia; 3 Universidad El Bosque, School of Dentistry, Unit of Basic Oral Investigation - UIBO, Bogotá, Colombia; 4 Department of Medical Sciences, University of Turin, Turin, Italy; 5 School of Biological Sciences, Universidad Pedagógica y Tecnológica de Colombia, Tunja, Colombia; OMICS, PERU

## Abstract

Triple-negative breast cancer (TNBC) remains the most aggressive breast cancer subtype, with the poorest prognosis among all forms of breast cancer. FOXA1 is a well-known pioneer factor in luminal breast cancer, however, despite its vital role in this tumor subtype, detailed information about FOXA1 expression and function in TNBC is notably lacking. This gap highlights the importance of exploring whether FOXA1 plays different roles across breast cancer subtypes. Accordingly, the present study aimed to assess the independent prognostic value of FOXA1 expression in a well-characterized cohort of 283 women with TNBC, evaluating its association with progression-free survival (PFS), overall survival (OS), and distant metastasis, and to determine whether this association is independent of the systemic treatment received. Initial analyses employed chi-square and t-tests to identify associations, followed by multivariate logistic and Cox regression models to determine independent predictors. Survival analyses were performed using Kaplan-Meier curves, with comparisons made using the log-rank test. The median age of patients was 69 years. FOXA1 positivity was significantly associated with grade II tumors (p < 0.001) and Ki-67 levels lower than 20% (p < 0.001). In multivariate analysis, FOXA1 was shown to be a poor prognostic factor for PFS (HRa = 1.68; 95% CI: 1.13–2.48; p = 0.009). Furthermore, in treatment-adjusted models, FOXA1 expression remained as an independent predictor of poor PFS (HRa = 1.96; 95% CI: 1.29–2.98; p = 0.001). These findings establish FOXA1 as an independent prognostic biomarker for disease progression in TNBC, with its adverse impact on PFS persisting regardless of the chemotherapeutic regimen administered.

## Introduction

Triple-negative breast cancer (TNBC) is a heterogeneous and clinically complex disease, characterized by the absence of well-defined therapeutic targets and by a more aggressive clinical behavior. Furthermore, TNBC is characterized by a higher recurrence rate, a marked tendency to metastasize, and consequently, lower overall survival compared to hormone-dependent tumors [1]. This underscores the importance of identifying and validating reliable prognostic and predictive biomarkers in this subgroup of patients. The transcription factor FOXA1 (Forkhead Box A1) is a particularly intriguing candidate. Historically, FOXA1 has been recognized as a key pioneering transcription factor for luminal differentiation and as a marker of good prognosis in luminal breast cancer [2–4]. However, evidence regarding its prognostic role in TNBC remains limited, consisting mostly of exploratory works, with preliminary clinical studies that mainly report associations without comprehensive multivariate analysis [5], and most of them, derived from methodologically heterogeneous studies [6]. Despite this, the literature suggests that positive FOXA1 expression in TNBC patients may be associated with increased disease-free survival [7–11]. These findings are promising but contrast with mechanistic studies in ER-positive endocrine-resistant breast cancer, where FOXA1 has been shown to promote metastasis [12]. While our findings are in TNBC, a subtype that does not receive endocrine therapy, this apparent discrepancy highlights the context-dependent role of FOXA1 across breast cancer subtypes. Regarding the role of FOXA1 in treatment response, a recent systematic review and meta-analysis in ER‑negative breast cancer (including seven studies [11,13–18]) explicitly noted that none of the included studies reported data on treatment response. Consequently, the predictive value of FOXA1 for chemotherapy or targeted therapy outcomes remains underexplored [6]. To date, no cohort study has specifically evaluated whether FOXA1 expression predicts treatment response in TNBC. Our study addresses this gap by providing the first cohort‑based assessment of FOXA1 as a potential prognostic biomarker in this context [6].

Therefore, although the available evidence suggests a protective effect, significant uncertainty remains. This highlights the need for (i) studies specifically designed for this purpose using homogeneous and well-defined cohort of breast cancer patients; (ii) apply a robust multivariate analysis that accounts for established prognostic factors and, importantly, incorporates treatment-related variables, which are often overlooked as potential confounders; and (iii) assess solid clinical outcomes, such as overall survival (OS) and progression-free survival (PFS). Accordingly, the present study aimed to assess the independent prognostic value of FOXA1 expression in a well-characterized cohort of TNBC patients, evaluating its association with PFS, OS, and distant metastasis, in addition to determining whether this association is independent of the systemic treatment received.

## Methodology

### Study design

We conducted a retrospective study including women aged ≥18 years diagnosed with TNBC between 2014–2024 at the Hospital Città della Salute e della Scienza di Torino, Turin, Italy, who had at least four years of clinical follow-up. Exclusion criteria comprised the absence of clinical follow-up, insufficient clinical data in medical records to determine the key clinicopathological variables required for the multivariable analyses (i.e., tumor size, nodal status, histological grade, and histotype), and biopsies unavailable for reanalysis or immunohistochemical verification. The study was conducted in accordance with the guidelines and regulations defined by the Research Ethics Committee for Human Biospecimen Utilization (Department of Medical Sciences (ChBU)) of the University of Turin. Given the retrospective design and absence of impact on clinical care, the informed consent requirement was waived. All data were anonymized to ensure confidentiality. Data was accessed and retrieved from the institutional database for research purposes on 20/12/2024.

### Sampling strategy and sample size

The case series was composed of consecutive cases, including all patients who met the eligibility criteria during the study period. To ensure adequate statistical power for the primary analysis, the sample size was calculated using OpenEpi software for comparing proportions in cohort studies [19]. A 95% confidence level and 80% statistical power were assumed, along with a balanced design (unexposed/exposed ratio of 1) and a mortality probability of 35% in the unexposed group, based on the findings of Beaubrun et al. 2022 [20]. Assuming a hazard ratio of 1.64 for the FOXA1-positive group, as reported by Fonseca-Benítez et al. 2025 [6], the minimum required sample size was estimated at 172 patients, with 86 FOXA1-positive and 86 FOXA1-negative individuals.

### Collection of clinical-pathological characteristics

Clinicopathological characteristics collected from medical reports for each patient included Age (years), Grade, pT, pN, Tumor Infiltrating Lymphocyte (TILs), Androgen Receptor (AR), CK5/6, p63, distant metastasis, date of recurrences and death. Categorization of these variables followed standard criteria and specific cut off were used for age (≥51 years) [21] and TILs < 10% - Low, 10–50%, Moderate, > 50% High [22]. Additionally, adjuvant and/or neoadjuvant systemic treatments were recorded and categorized as chemotherapy (CT), and neoadjuvant chemotherapy (NACT). It is important to remember that, regardless of the modality (monotherapy or combination), every chemotherapy regimen is based on the administration of specific drugs. Therefore, to capture their potential individual impact, chemotherapeutic agents were organized by their pharmacological class: taxanes, anthracyclines, alkylating agents, and platinum salts. Notably, the NACT administered in this cohort was composed of these same drugs, either in standard combinations or, in a subset of patients, as monotherapy or incomplete regimens, reflecting real-world clinical practice. For the primary analysis, NACT was assessed as a single binary variable (received vs. not received).

Finally, three main outcome variables were defined and calculated according to standard recommended criteria: PFS was defined as the time in months from histological diagnosis to disease progression, death from any cause, or, failing that, the date of the last follow-up (censorship). Patients without an event at the last follow-up were censored on that date and recorded as having no PFS. OS was the time in months from diagnosis to death from any cause. Patients alive at the end of the study were censored at their last confirmed contact date. Survival status was recorded as alive. Distant metastasis was a binary variable (Yes/No). Distant metastasis was defined as the documented presence of a metastatic lesion at a distant site during follow-up, based on histopathological confirmation and/or unequivocal imaging findings. Time to distant metastasis was calculated as the interval from diagnosis to the first documented metastasis [23].

### Immunohistochemical analysis

Immunohistochemistry (IHC) was performed on tissue microarray (TMA) sections [24] using an automated slide processing platform (Ventana BenchMark AutoStainer, Ventana Medical Systems, Tucson, AZ, USA) to validate diagnostic report findings. Immunohistochemical staining was performed using the following primary antibodies: prediluted rabbit monoclonal anti-ER (SP1, Ventana‑Roche), prediluted rabbit monoclonal anti-PR (1E2, Ventana‑Roche), polyclonal anti-HER2 (A0485, diluted 1:800; Dako, Denmark), mouse monoclonal anti-Ki67 (MIB1, diluted 1:50; Dako), and prediluted mouse monoclonal anti-FOXA1 (2F83, Ventana‑Roche). Detection was performed using the Ventana ultraView Universal DAB Detection Kit (Ventana‑Roche), with 3,3’‑diaminobenzidine (DAB) as the chromogen and hematoxylin as the counterstain. ER and PR positivity were defined in accordance with the ASCO/CAP guidelines (≥1% of tumor cells with nuclear staining). HER2 status was evaluated following ASCO/CAP 2007/2013 recommendations, and all equivocal cases (score 2+) were further assessed by fluorescence in situ hybridization (FISH). The Ki‑67 labelling index was determined using the MIB1 antibody with a cut‑off 20% for high proliferation, in accordance with the 2013 St. Gallen Consensus [25]. Positive and negative controls were included for each antibody assessment. A cutoff value of 1% or less was considered negative, while values of 1% or greater were considered positive for AR and FOXA1, consistent with previous studies [26,27]. CK5/6 and p63 positivity was defined as ≥1% nuclear or cytoplasmic staining [28]. IHC assessment was conducted by pathologists in a blinded manner, without knowledge of the patients’ clinical data and outcomes. All patients were confirmed as TNBC cases.

### Statistical analysis

The Shapiro-Wilk test was used to assess the normality of quantitative variables. Data were summarized as mean ± standard deviation (SD) for normally distributed variables or as median and interquartile range for non-normally distributed variables. The analysis evaluated comparability between groups defined by FOXA1 expression (positive Vs. negative) and examined crude associations between clinical-pathological variables and outcomes. For categorical variables, either the Pearson chi-square test or Fisher’s exact test was applied. Associations between FOXA1 expression and time-to-event outcomes were initially analyzed without adjustment. Survival curves for time to distant metastasis, PFS, and OS were estimated using the Kaplan-Meier method. Differences between groups were assessed using the log-rank test. Univariate Cox regression models quantified these associations, and results were presented as crude hazard ratios or unadjusted (HRc) and adjusted hazard ratios (HRa), with 95% confidence intervals (CI). For HRa, candidate variables were selected based on established clinical prognostic relevance or p-values less than 0.10 in the univariate analysis. A p-value less than 0.05 in the final multivariate model indicated a significant and independent prognostic factor. All analyses were performed using Stata version 14 software (StataCorp LP, College Station, TX, USA).

### External validation using web-based platforms

External validation was performed using the Kaplan-Meier Plotter platform (KM Plotter, https://kmplot.com/analysis/), a publicly available database integrating gene expression and clinical data from several breast cancer cohorts [29]. FOXA1 mRNA expression was analyzed using the probe 204667_at. The analysis was restricted to patients with a triple-negative phenotype (ER-negative, PR-negative, HER2-negative). To align with the immunohistochemical cutoff used in our cohort (FOXA1 0 vs ≥ 1), patients were dichotomized using the lower quartile of FOXA1 mRNA expression as the cutoff. Distant metastasis-free survival (DMFS), overall survival (OS), and relapse-free survival (RFS) were used as endpoints. Hazard ratios with 95% confidence intervals and log-rank p-values were calculated.

In addition, to assess the potential of FOXA1 as a predictive biomarker of chemotherapy response, we used the ROC Plotter platform (https://rocplot.org/), which integrates transcriptomic and clinical response data from breast cancer patients [30]. The analysis was restricted to TNBC/ER-negative patients treated with chemotherapy. The area under the curve (AUC), optimal cutoff, true positive rate (TPR), and true negative rate (TNR) were calculated to evaluate the predictive performance of FOXA1 mRNA expression for pathological response. Given the exploratory nature of this analysis, results were considered hypothesis-generating and interpreted in the context of the primary cohort findings.

## Results

### Clinical-pathological characteristics of patients

The study cohort consisted of 283 women with a mean age of 69.01 ± 14.49 years. The majority of tumors (87%) were histological grade III. Regarding tumor size, the lesions were similarly distributed between pT1 (46%) and pT2 (44%). Most patients (57%) had no lymph node involvement (pN0) at diagnosis. Ki-67 expression was high (>21%) in almost all cases (94%).

Non-special type carcinoma (NST) was the most frequent histological subtype, identified in 209 cases (73.9%), whereas less common subtypes like, apocrine, medullary-like, mucinous, among others accounted for less than 8% of total cases. Tumor Infiltrate lymphocytic (TILs) was assessed in 205 patients, where 115 (56.1%) were classified as low, 73 (35.6%) as moderate, and 17 (8.3%) as high (Table 1). With respect to FOXA1, the groups were comparable, 176 patients (62.2%) were classified as FOXA1 negative and 106 (37.8%) as FOXA1 positive (Fig 1). For baseline phenotype markers, AR expression was positive in 102 of 270 cases (37.8%), CK5/6 in 101 of 256 (39.5%), and p63 in 30 of 257 (11.7%). Therapeutically, more than half of the cohort (∼55%) received combined chemotherapy; taxane-based regimens (80%) were the most used, followed by anthracyclines (43.5%). During follow-up, 46,6% of the patients developed metastases, predominantly in the lungs, brain, and bones. A detailed summary of the characteristics is presented in **Table 1**.

**Table 1:** Clinical-pathological characteristics of patients with TNBC and treatment.

Characteristics	Description	n	%	IC 95%
**Age (years)**	< 50	29	10.2	(0.0 - 21.3)
	> 51	254	89.3	(86.0-93.5)
**Grade**	Grade II	37	13.3	(2.4 - 24.3)
	Grade III	241	86.7	(82.4 - 91.0)
**pT**	0	2	0.7	(0.0 - 12.4)
	pT1	130	46.3	(37.7 - 54.8)
	pT2	125	44.5	(35.8 - 53.2)
	pT3	17	6,0	(0.0 - 17.4)
	pT4	7	2.5	(0.0 - 14.0)
**pN**	pNX	17	6.1	(0.0 - 17.5)
	pN0	159	57.2	(49.5 - 64.9)
	pN1	10	3.6	(0.0 - 15.1)
	pN1a	48	17.3	(6.6 - 28.0)
	pN1b	3	1.1	(0.0 - 12.8)
	pN2	20	7.2	(0.0 - 18.5)
	pN3	2	0.7	(0.0 - 12.4)
	pN3a	18	6.5	(0.0 - 17.8)
	pN3c	1	0.4	(0.0 - 12.1)
**Ki-67**	< 20	18	6.4	(0.0 - 17.6)
	> 21	265	93.6	(90.7 - 96.6)
**Histotype**	NST	209	73.9	(67.9 - 79.8)
	ILC	13	4.6	(0.0 - 16.0)
	Apocrine	20	7.1	(0.0 - 18.3)
	Mucinous	8	2.8	(0.0 - 14.3)
	Medullary-like	16	5.7	(0.0 - 17.0)
	Papillary	3	1.1	(0.0 - 12.6)
	Micropapillary	4	1.7	(0.0 - 13.0)
	Metaplastic squamous	4	1.4	(0.0 - 13.0)
	Metaplastic	6	2.1	(0.0 - 13.6)
**TILs**	Low	115	56.1	(47.0 - 65.2)
	Moderate	73	35.6	(24.6 - 46.6)
	High	17	8.3	(0.0 - 21.4)
**AR**	<1	168	62.2	(54.9 - 69.6)
	≥1	102	37.8	(28.4 - 47.2)
**CK5/6**	<1	155	60.5	(52.9 - 68.2)
	≥1	101	39.5	(29.9 - 49.0)
**p63**	<1	227	88.3	(84.1 - 92.5)
	≥1	30	11.7	(0.2 - 23.2)
**FOXA1**	<1	176	62.2	(55.0 - 69.4)
	≥1	107	37.8	(28.6 - 47.0)
**Metastasis**	No	150	53.4	(45.4 - 61.4)
	Yes	131	46.6	(38.1 - 55.2)
**Multiple Metastasis**	No	125	96.2	(92.8 - 99.5)
	Yes	5	3.8	(0.0 - 20.7)
**Mortality**	Alive	209	73.9	(67.9 - 79.8)
	Died	74	26.1	(16.1 - 36.2)
**Treatment**				
	**Description**	**n**	**%**	**IC 95%**
**NACT**	No	226	79.9	(74.6 - 85.1)
	Yes	57	20.1	(9.7 - 30.6)
**Anthracyclines**	No	118	56.5	(47.5 -65.4)
	Yes	91	43.5	(33.4 - 53.7)
**Taxanes**	No	41	19.6	(7.5 - 31.8)
	Yes	168	80.4	(74.4 - 86.4)
**Antimetabolites**	No	167	79.9	(73.8 - 86.0)
	Yes	42	20.1	(8.0 - 32.2)
**Alkylating Agents**	No	145	69.4	(61. - 76.9)
	Yes	64	30.6	(19.3 - 41.9)

Categorical variables are reported as numbers and percentages (n (%)) and IC95% interquartile range. Abbreviations: AR, androgen receptor; CK5/6, cytokeratin 5/6; NACT, neoadjuvant chemotherapy; pN, pathological nodal stage; pT, pathological tumor stage; TILs, tumor-infiltrating lymphocytes.

**Fig 1:**
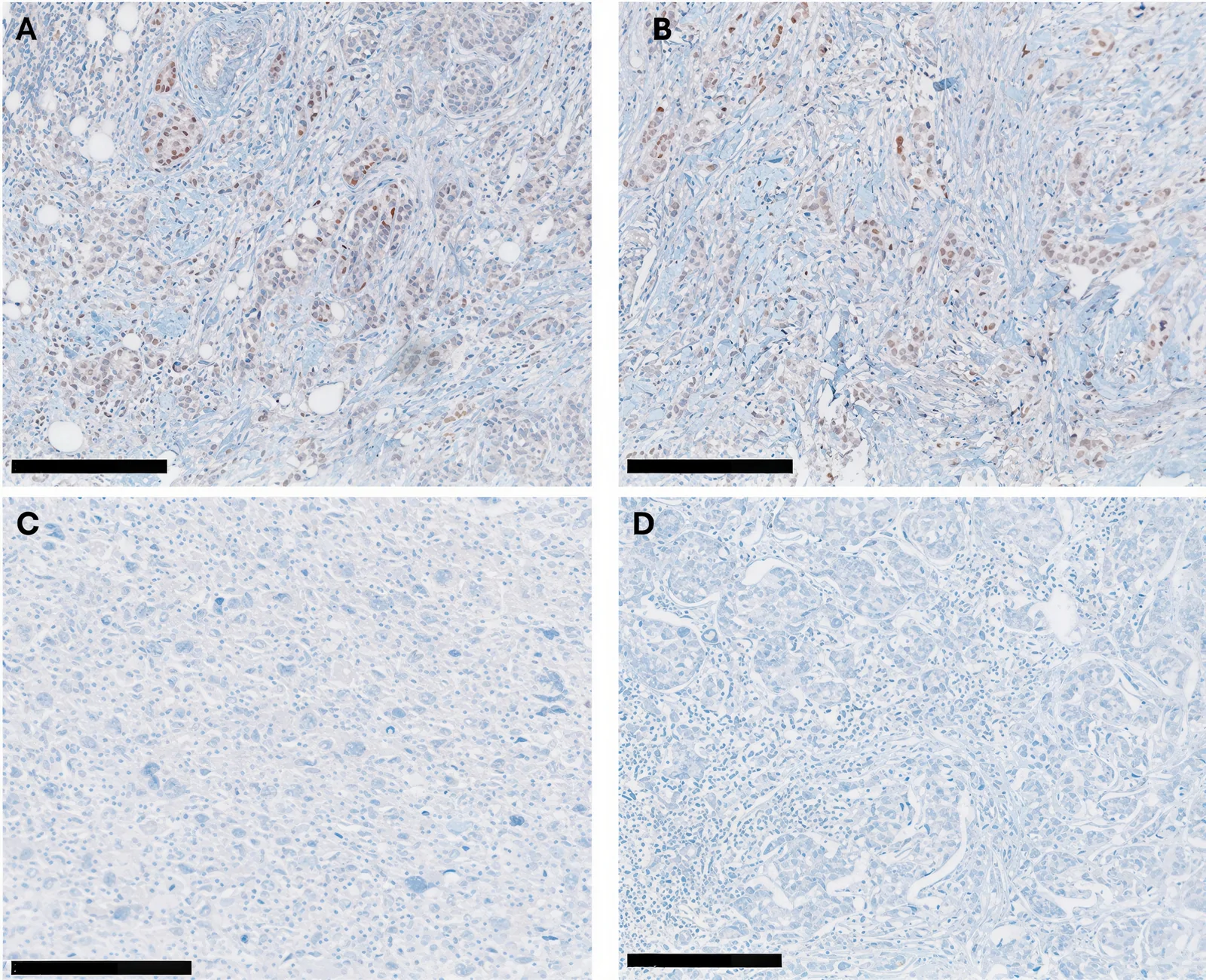
Expression of FOXA1 in human breast tumor tissue by immunohistochemistry. (A, B) TNBC with positive nuclear expression of FOXA1 (brown Chromogen – FOXA1 antibody; hematoxylin counterstain in blue). (C, D) TNBC with negative nuclear expression of FOXA1 (only hematoxylin counterstain in blue). Black bar 250 µM.

### Clinical‑pathological characteristics stratified by FOXA1 status

In Table 2 a comparison of baseline clinical-pathological characteristics of the cohort according to FOXA1 status is presented. The age distribution (≤50 years vs. > 51 years) was similar between the two groups (*p* = 0.697). Tumors with positive FOXA1 expression showed a significantly higher proportion of Grade II cases (24%) than the FOXA1-negative group (7%) (*p* < 0.001) and exhibited a significantly low proliferation index-Ki-67 < 20% (98%. *p* < 0.001), as well as higher incidence of distant metastasis (55%) compared with FOXA1-negative group (*p* = 0.025). Furthermore, the association between FOXA1 expression and histological type revealed a significantly different distribution across groups (*p* < 0.001). FOXA1 positivity was mainly observed in the apocrine and lobular (ILC) subtypes. Specifically, the apocrine subtype accounted for 17% of all FOXA1-positive tumors, compared to 1% in the negative group. In line with above, FOXA1 expression was also associated with AR positivity (*p* < 0.001).

**Table 2:** Comparison of baseline clinical-pathological characteristics of the cohort according to FOXA1 status.

Characteristics	Description	FOXA1				
		Negative		Positive		*p* value
		n	%	n	%	
**Age (Years)**	< 50	157	61	10	34	0.697
	> 51	19	65	97	38	
**Grade**	Grade II	12	7	25	24	<.001*
	Grade III	163	93	78	76	
**pT**	pT0	0	0	2	2	0.246
	pT1	79	45	51	48	
	pT2	83	47	42	40	
	pT3	10	6	7	7	
	pT4	3	2	4	4	
**Lymph node status**	pNX	9	5	8	8	0.245
	pN0	106	61	53	50	
	pN1	5	3	5	5	
	pN1a	27	16	21	20	
	pN1b	3	2	0	0	
	pN2	13	8	7	7	
	pN3	0	0	2	2	
	pN3a	10	6	8	8	
	pN3c	0	0	1	1	
**Ki-67**	< 20	3	2	15	14	<.001*
	> 21	173	98	92	86	
**Histotype**	NST	140	80	69	64	
	ILC	3	2	10	9	<.001*
	Apocrine	2	1	18	17	
	Mucinous	8	5	0	0	
	Medullary	14	8	2	2	
	Papillary	2	1	1	1	
	Micropapillary	1	1	3	3	
	Metaplastic squamous	3	2	1	1	
	Metaplastic	3	2	3	3	
**TILs**	Low	70	55	45	58	0.059
	Moderate	42	33	31	40	
	High	15	12	2	3	
**AR**	<1	131	78	37	36	<.001*
	≥1	36	22	66	64	
**CK5/6**	<1	85	54	70	71	0.008*
	≥1	72	46	29	29	
**p63**	<1	136	85	91	94	0.033*
	≥1	24	15	6	6	
**Metastasis**	No	103	59	47	45	0.025*
	Yes	73	41	58	55	
**Multiple Metastasis**	No	68	94	57	98	0.380
	Yes	4	6	1	2	
**OS**	No	133	76	76	71	0.399
	Yes	43	24	31	29	
**PFS**	No	83	47	37	35	0.044*
	Yes	93	53	69	65	
**Treatment**						
**NACT**	No	140	80	86	80	0.866
	Yes	36	20	21	20	
**Anthracyclines**	No	78	57	40	56	0.980
	Yes	60	43	31	44	
**Taxane**	No	26	19	15	21	0.693
	Yes	112	81	56	79	
**Antimetabolites**	No	107	78	60	85	0.234
	Yes	31	22	11	15	
**Alkylating Agents**	No	95	69	50	70	0.814
	Yes	43	31	21	30	

Baseline clinicopathological features were compared between groups with positive and negative FOXA1 expression. Categorical variables are reported as numbers and percentages (n (%)) and analyzed using the Pearson chi-square test or Fisher’s exact test. Continuous variables are presented as mean  or median (interquartile range) and compared using the Student’s t-test. Statistical significance (*) was defined as a p-value less than 0.05. Abbreviations: AR, androgen receptor; CK5/6, cytokeratin 5/6; NACT, neoadjuvant chemotherapy; pN, pathological nodal stage; pT, pathological tumor stage; TILs, tumor-infiltrating lymphocytes.

Analysis of basal markers revealed that CK5/6 and p63 were significantly more prevalent in FOXA1-negative tumors compared to FOXA1-positive tumors (p < 0.05). Regarding survival, no statistically significant difference in OS was observed between FOXA1-positive and FOXA1-negative patients (29% vs. 43%, respectively). However, the PFS rate was significantly higher in the FOXA1-positive group (65%) than in the FOXA1-negative group (53%) (p = 0.044). Moreover, chemotherapy regimens showed a comparable distribution between groups and no significant differences.

### Survival analysis according to FOXA1 status

Patients with FOXA1-positive disease had a median metastasis-free survival of 22 months (95% CI: 2–41), while those with FOXA1-negative disease had a median of 72.6 months (95% CI: 16–128). Although the survival curve suggests a higher risk in the FOXA1-positive group, the difference did not reach statistical significance (log-rank = 3.265; p = 0.071) (Fig 2A). For PFS (Fig 2B), patients in the FOXA1-positive group showed a significant worse prognosis, 37.7 months (95% CI: 20–55) compared with those in the FOXA1-negative group, 88.6 months (95% CI: 69–108) during the entire follow-up period (log-rank = 4.696; p = 0.032). Finally, the OS analysis did not reveal a statistically significant association between FOXA1 expression and mortality. The median OS was 45 months (95% CI: 37–52) for FOXA1-positive patients and 37 months (95% CI: 30–43) for FOXA1-negative patients (log-rank = 0.168; p = 0.0682) (Fig 2C).

**Fig 2:**
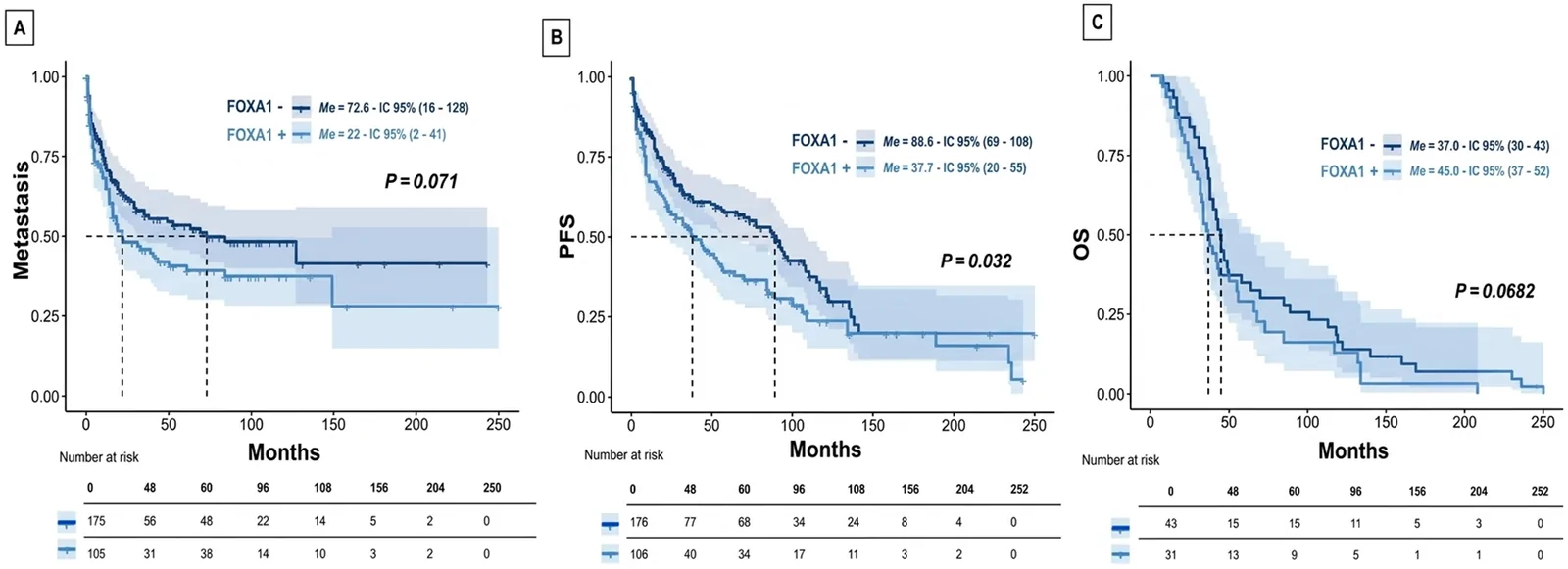
Kaplan-Meier curves comparing time to events between patients with TNBC and those with positive (FOXA1 + , light blue line) or negative (FOXA1-, dark blue line) FOXA1 expression. (A) Metastasis. (B) OS. (C) PFS. Each graph represent the number of patients at risk in each time interval for each group. The p-values correspond to the log-rank test comparing the two curves.

Additionally, univariate analyses were performed on baseline variables related to the outcomes, which are summarized in S1, S2, and S3 Tables in S1 File. Significant associations with several variables were identified for metastasis, OS and PFS. Factors associated with improved or worsened PFS included pT (p = 0.002) and NACT treatment (p = 0.003) (S2 Table in S1 File). OS (S3 Table in S1 File) and Metastasis (S1 Table in S1 File) were significantly associated with pT (p = 0.016); pN (p =<,001) and NACT (p = 0.03).

### Evaluation of the independent prognostic value of FOXA1 using Cox regression models

A multivariate logistic regression model was developed to assess whether FOXA1 may be associated as an independent prognostic factor for distant metastasis, PFS, and OS. To evaluate the association between FOXA1 and survival outcomes, three Cox proportional hazards models were constructed. First, univariable models were fitted for each variable to obtain crude hazard ratios (HRc). Subsequently, two adjusted multivariate models were specified. The first adjusted model (HRa) included clinicopathological variables with established prognostic value: age, histological grade, pT stage, and Ki-67 proliferation index. The second adjusted model incorporated the chemotherapy received, to account for potential treatment selection bias. Both HRc and adjusted HRa are presented in the main results table to allow assessment of confounding, with the multivariate models representing the primary analysis.

The results of the analysis for distant metastasis and clinical features are presented in Table 3. Following multivariate adjustment, FOXA1 expression did not demonstrate an independent or statistically significant association with distant metastasis risk (p > 0.05). In contrast, when tumor-infiltrating lymphocytes (TILs) were analyzed as a continuous variable, a significant association with distant metastasis was observed in both models (HRc = 2.54; 95% CI: 1.80–3.59; HRa = 2.62; 95% CI: 1.81–3.80; p < 0.001). This finding indicates that a higher percentage of TILs was independently associated with an increased risk of developing distant metastases in this cohort. In the final treatment-adjusted model (Table 4), treatment with alkylating agents was the sole variable remaining statistically significant (HRa = 0.51; 95% CI: 0.30–0.88; p = 0.016), indicating an independent protective association.

**Table 3:** Multivariate Cox regression analysis for metastasis and clinical-pathologic characteristics.

Variable / Description		Metastasis		*p* Value
		HR unadjusted (HRc) (IC 95 %)	HR adjusted (HRa) (IC 95 %)	
**FOXA1**	**<1**	**≥1**	**≥1**	**0.895**
	**≥1**	0.98 (0.69 - 1.39)	1.03 (0.65 - 1.61)	
**Age (Years)**	**< 50**	**> 51**	**> 51**	**0.776**
	**> 51**	1.05 (0.61 - 1.78)	1.08 (0.63 - 1.84)	
**Grade**	**Grade II**	**Grade III**	**Grade III**	**0.801**
	**Grade III**	1.19 (0.69 - 1.85)	0.92 (0.49 - 1.72)	
**pT**	**pT0**	**pT4**	**pT4**	**0.216**
	**pT4**	0.85 (0.65 - 1.11)	0.83 (0.63 - 1.10)	
**Ki-67**	**< 20**	**> 21**	**> 21**	**0.491**
	**> 21**	1.34 (0.65 - 2.77)	1.36 (0.56 - 3.28)	
**AR**	**<1**	**≥1**	**≥1**	**0.847**
	**≥1**	0.93 (0.65 - 1.33)	0.95 (0.61 - 1.49)	
**TILs**	**Low**	**High**	**High**	0.000*
	**High**	2.54 (1.80 – 3.59)	2.62 (1.81 – 3.80)	

For each variable, the table presents the unadjusted (crude) hazard ratio (HRc) with its 95% confidence interval (CI) obtained from univariate Cox regression, alongside the adjusted hazard ratio (HRa) with its 95% CI derived from a multivariate Cox model. The multivariate model included age, histological grade, pT stage, Ki-67 proliferation index, AR and TILs. The p-value corresponds to the Wald test from the adjusted (multivariate) model. Statistical significance (*) was defined as a p-value less than 0.05. Abbreviations: HR, hazard ratio; CI, confidence interval;; pT, pathological tumor stage; AR, androgen receptor; TILs, tumor-infiltrating lymphocytes.

**Table 4:** Multivariate Cox regression analysis for metastasis and treatments.

Variable / Description		Metastasis		*p* Value
		HR unadjusted (HRc) (IC 95 %)	HR adjusted (HRa) (IC 95 %)	
**FOXA1**	**<1**	**≥1**	**≥1**	**0.383**
	**≥1**	0.98 (0.69 - 1.39)	0.83 (0.55 - 1.25)	
**NACT**	**No**	**Yes**	**Yes**	**0.206**
	**Yes**	0.75 (0.51 - 1.11)	0.76 (0.49 - 1.16)	
**Anthracycline**	**No**	**Yes**	**Yes**	**0.076**
	**Yes**	1.12 (0.76 - 1.66)	1.52 (0.95 - 2.44)	
**Taxanes**	**No**	**Yes**	**Yes**	**0.975**
	**Yes**	1.00 (0.63 - 1.59)	1.01 (0.53 - 1.90)	
**Antimetabolites**	**No**	**Yes**	**Yes**	**0.619**
	**Yes**	0.98 (0.63 - 1.52)	1.17 (0.62 - 2.19)	
**Alkylating** **agents**	**No**	**Yes**	**Yes**	0.016*
	**Yes**	0.69 (0.45 - 1.07)	0.51 (0.30 - 0.88)	

For each variable, the table presents the unadjusted (crude) hazard ratio (HRc) with its 95% confidence interval (CI) obtained from univariate Cox regression, alongside the adjusted hazard ratio (HRa) with its 95% CI derived from a multivariate Cox model. The multivariate model included systemic treatment administered. The p-value corresponds to the Wald test from the adjusted (multivariate) model. Statistical significance (*) was defined as a p-value less than 0.05. Abbreviations: HR, hazard ratio; CI, confidence interval; neoadjuvant chemotherapy, NACT.

Regarding PFS, FOXA1 was associated with a worse prognosis, reflected in a higher risk of progression or death, independent of the other variables in the model (HRa = 1.68; 95% CI: 1.13–2.48; p = 0.009). This behavior was similar to that observed for other covariate, such as pT stage (HRa = 2.02; 95% CI: 1.32–2.26; p < 0.001) (Table 5). When treatments were evaluated into the multivariate model, FOXA1 maintained its independent prognostic value for PFS (HRa = 1.96; 95% CI: 1.29–2.98; p = 0.001). Furthermore, the only group of chemotherapeutic agents that maintained statistical significance was that of antimetabolites (HRa = 1.63; 95% CI: 1.01–2.63; p = 0.042) (Table 6).

**Table 5:** Multivariate Cox regression analysis for PFS and clinical-pathologic characteristics.

Variable	Description	PFS		p value
		HR unadjusted (HRc) (IC 95 %)	HR adjusted (HRa) (IC 95 %)	
**FOXA1**	**<1**	**≥1**	**≥1**	0.009*
	**≥1**	1.37 (1.00 - 1.88)	1.68 (1.13 - 2.48)	
**Age (Years)**	**< 50**	**> 51**	**> 51**	0.033*
	**> 51**	0.59 (0.36 - 0.97)	1.66 (0.98 - 2.81)	
**Grade**	**Grade II**	**Grade III**	**Grade III**	**0.287**
	**Grade III**	0.90 (0.57 - 1.44)	0.74 (0.43 - 1.27)	
**pT**	**pT0**	**pT4**	**pT4**	0.000*
	**pT4**	4.08 (0.45 - 36.77)	2.02 (1.32 - 2.26)	
**Ki-67**	**< 20**	**> 21**	**> 21**	**0.143**
	**> 21**	1.42 (0.72 - 2.82)	1.79 (0.81 - 3.93)	
**AR**	**<1**	**≥1**	**≥1**	**0.549**
	**≥1**	1.06 (0.77 - 1.47)	0.89 (0.62 - 1.28)	
**TILs**	**Low**	**High**	**High**	**0.297**
	**High**	0.75 (0.56 – 1.00)	0.85 (0.63 – 1.14)	

For each variable, the table presents the unadjusted (crude) hazard ratio (HRc) with its 95% confidence interval (CI) obtained from univariate Cox regression, alongside the adjusted hazard ratio (HRa) with its 95% CI derived from a multivariate Cox model. The multivariate model included age, histological grade, pT stage, Ki-67 proliferation index, AR and TILs. The p-value corresponds to the Wald test from the adjusted (multivariate) model. Statistical significance (*) was defined as a p-value less than 0.05. Abbreviations: HR, hazard ratio; CI, confidence interval; pT, pathological tumor stage; AR, androgen receptor; TILs, tumor-infiltrating lymphocytes; PFS, progression-free survival.

**Table 6:** Multivariate Cox regression analysis for PFS and Treatments.

Variable	Description	PFS		*p* value
		HR unadjusted (HRc) (IC 95 %)	HR adjusted (HRa) (IC 95 %)	
**FOXA1**	**<1**	**≥1**	**≥1**	0.001*
	**≥1**	0.98 (0.69 - 1.39)	1.96 (1.29 - 2.98)	
**NACT**	**No**	**Yes**	**Yes**	**0.200**
	**Yes**	1.25 (0.85 - 1.83)	1.38 (0.84 - 2.26)	
**Anthracycline**	**No**	**Yes**	**Yes**	**0.919**
	**Yes**	0.99 (0.99 - 1.00)	1.05 (0.46 - 2.32)	
**Taxane**	**No**	**Yes**	**Yes**	**0.638**
	**Yes**	0.99 (0.99 - 1.00)	0.87 (0.50 - 1.51)	
**Antimetabolites**	**No**	**Yes**	**Yes**	0.042*
	**Yes**	1.60 (1.06 - 2.42)	1.63 (1.01 - 2.63)	
**Alkylating** **agents**	**No**	**Yes**	**Yes**	**0.153**
	**Yes**	0.99 (0.99 - 1.00)	1.50 (0.86 - 2.62)	

For each variable, the table presents the unadjusted (crude) hazard ratio (HRc) with its 95% confidence interval (CI) obtained from univariate Cox regression, alongside the adjusted hazard ratio (HRa) with its 95% CI derived from a multivariate Cox model. The multivariate model included systemic treatment administered. The p-value corresponds to the Wald test from the adjusted (multivariate) model. Statistical significance (*) was defined as a p-value less than 0.05. Abbreviations: HR, hazard ratio; CI, confidence interval; neoadjuvant chemotherapy, NACT; pT, pathological tumor stage; PFS, progression-free survival.

Finally, in the context of OS, FOXA1 expression showed no association with OS (HRa = 0.97; 95% CI: 0.51–1.87; p = 0.946). Unlike other clinical features, higher TIL levels, analyzed as a continuous variable, was significantly associated with improved OS in the adjusted model (HRa = 0.50; 95% CI: 0.31–0.81; p = 0.005) (Table 7). This finding suggests that, within this cohort, a greater immune infiltrate confers a survival advantage. Interestingly, the administration of NACT was independently associated with OS (HRa = 2.57; 95% CI: 1.10–5.56; p = 0.028), which is probably due to its use in tumors with worse baseline prognosis. (Table 8).

**Table 7:** Multivariate Cox regression analysis for OS Clinical-pathologic variables.

Variable	Description	OS		*p* Value
		HR unadjusted (HRc) (IC 95 %)	HR adjusted (HRa) (IC 95 %)	
**FOXA1**	**<1**	**≥1**	**≥1**	**0.946**
	**≥1**	1.04 (0.65 - 1.68)	0.97 (0.51 - 1.87)	
**Age (Years)**	**< 50**	**> 51**	**> 51**	**0.885**
	**> 51**	0.91 (0.41 - 2.01)	0.93 (0.39 - 2.22)	
**Grade**	**Grade II**	**Grade III**	**Grade III**	**0.119**
	**Grade III**	1.65 (0.78 - 3.50)	1.87 (0.84 - 4.13)	
**pT**	**pT0**	**pT4**	**pT4**	**0.376**
	**pT4**	1.11 (0.78 - 1.58)	1.19 (0.80 - 1.77)	
**Ki-67**	**< 20**	**> 21**	**> 21**	**0.471**
	**> 21**	0.86 (0.31- 2.38)	0.67 (0.22 - 1.98)	
**AR**	**<1**	**≥1**	**≥1**	**0.373**
	**≥1**	1.14 (0.71 - 1.85)	1.33 (0.70 - 2.49)	
**TILs**	**Low**	**High**	**High**	**0.005**
	**High**	0.59 (0.39 – 0.89)	0.50 (0.31 – 0.81)	

For each variable, the table presents the unadjusted (crude) hazard ratio (HRc) with its 95% confidence interval (CI) obtained from univariate Cox regression, alongside the adjusted hazard ratio (HRa) with its 95% CI derived from a multivariate Cox model. The multivariate model included age, histological grade, pT stage, Ki-67 proliferation index, AR and TILs. The p-value corresponds to the Wald test from the adjusted (multivariate) model. Statistical significance (*) was defined as a p-value less than 0.05. Abbreviations: pT, pathological tumor stage; AR, androgen receptor; TILs, tumor-infiltrating lymphocytes; OS, overall survival.

**Table 8:** Multivariate Cox regression analysis for OS and treatments.

Variable	Description	OS		*p* Value
		HR unadjusted (HRc) (IC 95%)	HR adjusted (HRa) (IC 95%)	
**FOXA1**	**<1**	**≥1**	**≥1**	**0.030**
	**≥1**	1.04 (0.65 - 1.68)	0.43 (0.20 - 0.91)	
**NACT**	**No**	**Yes**	**Yes**	**0,028**
	**Yes**	1.50 (0.84 - 2.68)	2.47 (1.10 - 5.56)	
**Anthracycline**	**No**	**Yes**	**Yes**	**0,083**
	**Yes**	1.39 (0.76 - 2.56)	2.11 (0.90 - 4.91)	
**Taxane**	**No**	**Yes**	**Yes**	**0,197**
	**Yes**	1.49 (0.76 - 2.93)	1.84 (0.72 - 4.65)	
**Antimetabolites**	**No**	**Yes**	**Yes**	**0,365**
	**Yes**	1.00 (0.53 - 1.90)	1.55 (0.59 - 4.04)	
**Alkylating agents**	**No**	**Yes**	**Yes**	**0,200**
	**Yes**	0.73 (0.39 - 1.36)	0.55 (0.22 - 1.36)	

For each variable, the table presents the unadjusted (crude) hazard ratio (HRc) with its 95% confidence interval (CI) obtained from univariate Cox regression, alongside the adjusted hazard ratio (HRa) with its 95% CI derived from a multivariate Cox model. The multivariate model included systemic treatment administered. The p-value corresponds to the Wald test from the adjusted (multivariate) model. Statistical significance (*) was defined as a p-value less than 0.05. Abbreviations: HR, hazard ratio; CI, confidence interval; neoadjuvant chemotherapy, NACT; OS, overall survival.

### External validation using web-based platforms

To explore whether the prognostic direction observed in our cohort could be replicated in independent datasets, we performed an exploratory analysis using the Kaplan-Meier Plotter platform, a meta-cohort integrating data from multiple public repositories, including GEO, TCGA, and METABRIC. In TNBC/ER-negative patients, high FOXA1 mRNA expression showed trends toward worse DMFS (n = 424; HR = 1.47; 95% CI: 0.96–2.25; p = 0.075) and worse RFS (n = 534; HR = 1.64; 95% CI: 1.12–2.38; p = 0.0097), both consistent in direction with our cohort findings. Notably, OS showed no significant association with FOXA1 expression (n = 201; HR = 1.1; 95% CI: 0.54–2.23; p = 0.79). However, the lower bound of the confidence interval (0.54) suggests a trend toward a favorable outcome in patients with high FOXA1 expression, mirroring the paradoxical dissociation between PFS and OS observed in our cohort (HR = 1.04). The opposite direction observed for OS, although non-significant, may reflect the influence of post-progression therapies or the limited number of OS events in this subgroup. The significant association with RFS, together with the consistent direction observed for DMFS, supports the adverse prognostic role of FOXA1 positivity in TNBC observed in our cohort (Supplementary S1 Fig in S1 File).

In the exploratory analysis using ROC Plotter, FOXA1 mRNA expression showed a modest but statistically significant predictive performance for chemotherapy response in TNBC/ER-negative patients (AUC = 0.615; p = 0.00444; TPR = 0.66; TNR = 0.55). While the AUC indicates limited predictive utility as a single biomarker, these findings suggest that FOXA1 may have some capacity to distinguish responders from non-responders (Supplementary S2 Fig in S1 File).

## Discussion

This study demonstrates that FOXA1 expression plays a complex and context-dependent prognostic role in breast cancer. Notably, in our TNBC cohort, FOXA1 positivity was associated with clinicopathological features, like a significantly lower Ki-67 proliferation index and a trend toward lower TILs infiltration (p = 0.059). These findings align with a less proliferative and potentially immune-evasive tumor phenotype. Furthermore, FOXA1-positive tumors exhibited a numerically higher incidence of distant metastases and significantly shorter PFS. To note, this unfavorable association persisted after adjusting for clinicopathological variables and, crucially, remained independent of the chemotherapeutic treatment received, underscoring an unfavorable prognosis for disease progression that is not attributable to differences in therapy. Regarding the high proportion of grade III tumors in our cohort (87%), this distribution reflects the well-established aggressive nature of TNBC rather than a selection bias. Importantly, FOXA1’s prognostic effect remained independent of grade in multivariate analysis, confirming that its adverse impact on PFS is not merely a surrogate of high-grade histology.

This observation is consistent with previous reports linking FOXA1 expressions to adverse outcomes in TNBC. Govindan et al. identified a molecular signature characterized by high mRNA expression levels of AR, FOXA1 and GATA3, that was strongly associated with poor prognosis, despite low Ki67 levels [31]. Similar to the low Ki-67 reported in some FOXA1-positive LAR tumors, our cohort showed a significant association between FOXA1 positivity, AR positivity and Ki-67 < 20% (p < 0.001). Our findings suggest that in a subset of TNBC, FOXA1 may be associated with a less proliferative phenotype, which indicate that the relationship between FOXA1 and proliferation is context-dependent and probably vary across TNBC molecular subtypes [32]. Further studies are needed to clarify whether FOXA1 drives proliferation directly or serves as a marker of a specific TNBC subtype with intrinsically higher proliferative activity.

Together with our findings, where FOXA1-positive tumors display low Ki-67 and unfavorable PFS, these data suggest that FOXA1’s adverse prognostic impact in TNBC may be independent of the proliferative status and could be driven by distinct molecular contexts, including AR co-expression and immune microenvironment features such as TILs. In this regard, analysis of TILs revealed that, as a continuous variable, higher TIL levels were associated with an increased risk of distant metastasis (HRa = 2.62; 95% CI: 1.81–3.80), while also demonstrating a strong association with improved OS (HRa = 0.50; 95% CI: 0.31–0.81).This dissociation mirrors our findings with FOXA1 and suggests that, in this cohort, a more immune-inflamed tumor microenvironment may facilitate early dissemination, potentially through matrix metalloproteinase-mediated extracellular matrix remodeling and epithelial-to-mesenchymal transition induced by inflammatory cytokines [33] while simultaneously enhancing sensitivity to post-progression therapies. This interpretation aligns with evidence that TIL-high TNBCs are more likely to respond to salvage chemotherapy and immunotherapy [34]. Although our data does not allow for phenotypic characterization of the immune infiltrate, this finding underscores the complexity of the immune contexture in TNBC and highlights the need to consider both progression and mortality endpoints when evaluating immune biomarkers.

A key strength of the present study lies in the robustness of the observed association between FOXA1 positivity and adverse PFS. Notably, this association not only persisted but was strengthened after adjustment for major clinicopathological confounders, including age, histological grade, and Ki-67 index, indicating that the prognostic impact of FOXA1 is not merely a surrogate for a more proliferative or poorly differentiated phenotype. Furthermore, the prognostic value remained statistically significant even after controlling the type of adjuvant treatment received. This finding indicates that the adverse effect of FOXA1 on disease progression is independent of the specific therapeutic regimen administered, thereby establishing FOXA1 as an independent prognostic biomarker in TNBC. The initial evaluation of individual drug classes, beyond the clinical label of NACT, ensured that no single pharmacological group was responsible for or confounding the observed association.

These results differ notably from the established view of FOXA1 as a marker of favorable prognosis in TNBC [35]. In luminal tumors, elevated FOXA1 expression has been consistently associated with delayed recurrences and improved long-term outcomes [36]. In contrast, the role of FOXA1 in TNBC subtypes is less defined. Some studies in TNBC have similarly reported adverse prognostic associations [17], indicating that FOXA1 function is highly context dependent. Our findings align with these reports regarding progression-related endpoints and underscore a subtype-specific switch in prognostic behavior.

A particularly relevant aspect of our data is the strong association between FOXA1 positivity and AR expression (p < 0.001), with the apocrine subtype accounting for 17% of FOXA1-positive tumors compared to only 1% in the FOXA1-negative group. This observation is consistent with previous studies by our group and others, which have shown a strong correlation between AR and FOXA1 expression in TNBC, defining a distinct molecular apocrine or LAR subtype [4,37]. In this context, FOXA1 may function as a pioneer factor facilitating AR-driven transcription [38]. Future prospective studies with larger cohorts are needed to validate these associations and to evaluate the independent or synergistic prognostic effects of FOXA1 and AR, as well as their potential utility in guiding AR-targeted therapies.

A potential mechanistic basis for the adverse effect of FOXA1 on PFS and metastasis in TNBC may lie in its function as a pioneer transcription factor capable of activating pro-metastatic transcriptional programs independently of ER [12,39]. In pancreatic cancer, FOXA1 has been shown to drive enhancer reprogramming that promotes invasion and metastasis [40], establishing a paradigm in which FOXA1 itself acts as a direct promoter of metastatic capacity. In the TNBC context, emerging evidence supports an analogous role: FOXA1 overexpression in TNBC cells activates downstream targets such as DCTPP1 that sustain tumor progression and metastatic behavior [41]. Furthermore, pan-cancer genomic analyses have demonstrated that FOXA1 amplification is associated with enhanced metastatic potential and decreased survival [42]. These observations are consistent with prior reports indicating that, among TNBC, tumors with a specific luminal androgen receptor (LAR) phenotype, which are typically FOXA1-positive, show a higher rate of relapses and deaths despite displaying low-grade features and reduced Ki-67 [38]. Collectively, these findings support a model in which sustained FOXA1 expression in TNBC contributes to a pro-metastatic phenotype, potentially explaining the adverse PFS and higher metastatic propensity observed in our FOXA1-positive cohort.

Furthermore, our finding that FOXA1-positive tumors exhibited decreased TILs aligns with the report by Mangia et al. (2019), who observed low TILs in FOXA1-positive TNBC (p = 0.028) and noted that the combination of positive FOXA1 and high TILs predicted better PFS [10]. Though the specific interaction was not replicated here, the consistent trend toward reduced immune infiltration reinforces the notion of a prognostically unfavorable tumor microenvironment in FOXA1-positive TNBC. Mechanistically, FOXA1 has been shown to transcriptionally repress IL-6 by binding to its promoter and preventing NF-κB recruitment [16], suggesting that FOXA1 expression may lead to a less inflamed tumor microenvironment, which is consistent with the reduced TIL infiltration observed in FOXA1-positive tumors in our cohort. This mechanism provides a plausible link between FOXA1 expression and the immune-evasive microenvironment in TNBC.

Moreover, the association between FOXA1 and tumor proliferation in breast cancer is complex and context dependent. Studies have identified a subgroup of tumors with high FOXA1 expression, low Ki-67, and AR positivity that paradoxically exhibits aggressive clinical behavior, including lower recurrence-free and overall survival [43]. This discrepancy suggests that the prognostic value of FOXA1 cannot be explained solely by its relationship with proliferation but is modulated by the molecular context, particularly AR co-expression. In our cohort, we also observed a lower Ki-67 trend in FOXA1 + tumors, which may reflect an unclear mechanism between FOXA1 and cell-cycle regulation, similar to that described in LAR tumors subtype [38]. Future studies with deeper molecular characterization will be necessary to validate this hypothesis.

In addition, our analysis also identified NACT and the use of alkylating agents as factors associated with worse outcomes. Rather than suggesting a detrimental effect of these treatments per se, this likely reflects indication bias, as more intensive regimen or the use of alternative therapies (e.g., immunotherapy) to patients with higher-risk diseases. Notably, evaluating individual drug classes allowed us to confirm that the adverse prognostic effect of FOXA1 was not driven by any single pharmacological group, thereby reinforcing the robustness of its independent prognostic value. Indeed, the association between FOXA1 and PFS remained stable after adjustment for treatment variables, supporting its role as an independent prognostic factor. Although the therapeutic landscape of these tumors has now evolved toward alternative therapies and a predominantly neoadjuvant approach, our findings indicate that the prognostic value of FOXA1 is maintained. In addition, its association with AR further underscores the clinical and therapeutic relevance of the LAR subtype, reinforcing the rationale for tailored treatment strategies in this specific patient population.

A notable finding was the non-significant trend toward better OS observed in FOXA1-positive patients (median 45.0 vs. 37.0 months; p = 0.0682), despite their clearly inferior PFS. This pattern is illustrated in Fig 2B, where the OS curve for FOXA1-positive patients falls below that of FOXA1-negative patients during the later follow-up period. One possible explanation is that FOXA1-positive tumors, while more susceptible to early progression, may demonstrate increased sensitivity to post-progression salvage therapies. This is supported by evidence from breast cancer patients who progressed during NACT and subsequently responded well to salvage treatment, achieving promising survival outcomes despite their initially poor prognosis [44]. The dissociation between PFS and OS is a well-recognized phenomenon in oncology, where post-progression therapies can attenuate observable OS differences [45]. Nevertheless, this observation regarding OS does not detract from the primary finding: FOXA1 positivity is a robust and independent predictor of early disease progression in this cohort. Further mechanistic studies are necessary to clarify the basis of the dissociation between PFS and OS.

Finally, to explore whether the prognostic and predictive patterns observed in our cohort could be replicated in independent datasets, we performed exploratory validations using KM Plotter and ROC Plotter. In KM Plotter, the effect direction for RFS and DMFS was consistent with our cohort findings, with higher FOXA1 expression correlating with poorer outcomes (RFS: HR = 1.64; p = 0.0097). This consistency across independent datasets, despite differences in measurement platforms and patient populations, suggests that the adverse prognostic value of FOXA1 in TNBC is a reproducible biological phenomenon rather than a cohort-specific artifact. Notably, the co-expression of AR and FOXA1 observed in our cohort is consistent with the molecular apocrine/LAR subtype described in the literature, which has been associated with worse outcomes and resistance to conventional chemotherapy [46]. The predictive analysis using ROC Plotter further supports this, showing a modest but statistically significant ability of FOXA1 to predict chemotherapy response (AUC = 0.615; p = 0.00444). Together, these findings underscore the need for prospective validation of FOXA1 in protein-based cohorts and highlight its potential as a biomarker beyond the luminal context.

### Strengths and limitations

This study demonstrates several methodological strengths. The predetermined sample size (n = 283) was calculated using OpenEpi and exceeded the minimum required to detect clinically relevant differences. This ensured adequate statistical power for the primary analysis. The cohort was sourced from referral center and provided high-quality clinical, pathological, and long-term follow-up data spanning at least four years. FOXA1 expression was centrally assessed via immunohistochemistry. Laboratory staff were blinded to clinical outcomes to minimize information bias. Comprehensive statistical analyses, including both univariate and multivariate models, were conducted to identify independent prognostic factors. These analyses are adjusted for key clinical and treatment variables.

Despite these strengths, several limitations should be acknowledged. As a retrospective, single-center study, the findings may have limited generalizability and may be subject to selection bias. First, type of therapy and its timing are now different in this tumor setting; consequently, the role of FOXA1 will need to be reassessed in more recent cohorts that could be more consistent with current clinical practice. Second, the cohort comprised all TNBC tumors subtypes, however, the sample size, while adequate for primary analyses, was insufficient for detailed subgroup or treatment-specific analyses. FOXA1 positivity was defined as nuclear staining of at least 1%, a threshold that may differ from those used in other studies and could contribute to variability in reported results. Finally, the absence of an external validation cohort necessitates confirmation of these findings in future prospective multicenter studies.

## Conclusion

The present study establishes FOXA1 as an independent prognostic biomarker for disease progression in TNBC, with its adverse impact on PFS persisting regardless of clinical-pathological variables and even chemotherapeutic regimen. The FOXA1-AR association underscores the clinical relevance of the LAR subtype. TILs showed a dual prognostic role: higher levels were associated with increased risk of distant metastasis but also with improved OS, reflecting the complex interplay among immune infiltration, dissemination, and treatment sensitivity. These findings challenge the paradigm of FOXA1 as exclusively a luminal marker of good prognosis and support its utility for identifying TNBC patients at higher risk of early progression. Prospective validation with detailed molecular and immune characterization is warranted to confirm FOXA1 as a clinically actionable biomarker.

## Supporting information

S1 File S1 Table. Clinical-pathological characteristics compared with metastasis. S2 Table. Clinical-pathological features compared with Progression-Free Survival. S3 Table. Clinical-pathological characteristics compared with Overall Survival. S1 Fig. External validation of FOXA1 prognostic value using KM Plotter. Kaplan-Meier curves. S2 Fig. Predictive performance of FOXA1 mRNA expression for chemotherapy response in TNBC/ER-negative patients using ROC Plotter.
(DOCX)
