# Supplementary material for: FOXA1 expression and survival outcomes in triple-negative breast cancer: A retrospective cohort study
Source: PLoS One. 2026 Nov 13;21(11):e0362480. doi: 10.1371/journal.pone.0362480 (PMC13766281; doi:10.1371/journal.pone.0362480)
Supplement: Supplementary file 1 [file pone.0362480.s001.docx]

**Supplementary Material**

**Table S1. Clinical-pathological characteristics compared with metastasis.**

| **Variable/Description** | | **Metastasis** | | | |  |
| --- | --- | --- | --- | --- | --- | --- |
|  |  | **No** | | **Yes** | |  |
|  |  | **n** | **%** | **n** | **%** | ***p* value** |
| **Age (Years)** | **< 50** | 13 | 8.0 | 16 | 12.2 | 0.311 |
|  | **> 51** | 139 | 91.4 | 115 | 87.8 |  |
| **Grade** | **Grade II** | 18 | 12.2 | 19 | 14.6 | 0,548 |
|  | **Grade III** | 130 | 87.8 | 111 | 85.4 |  |
| **pT** | **pT0** | 2 | 1.3 | 0 | 0.0 | <,001* |
|  | **pT1** | 81 | 54.0 | 48 | 36.9 |  |
|  | **pT2** | 58 | 38.7 | 67 | 51.5 |  |
|  | **pT3** | 7 | 4.7 | 10 | 7.7 |  |
|  | **pT4** | 2 | 1.3 | 5 | 3.8 |  |
| **pN** | **pNX** | 11 | 7.5 | 6 | 4.6 | 0,733 |
|  | **pN0** | 128 | 87.7 | 29 | 22.3 |  |
|  | **pN1** | 1 | 0.7 | 9 | 6.9 |  |
|  | **pN1a** | 6 | 4.1 | 42 | 32.3 |  |
|  | **pN1b** | 0 | 0.0 | 3 | 2.3 |  |
|  | **pN2** | 0 | 0.0 | 20 | 15.4 |  |
|  | **pN3** | 0 | 0.0 | 2 | 1.5 |  |
|  | **pN3a** | 0 | 0.0 | 18 | 13.8 |  |
|  | **pN3c** | 0 | 0.0 | 1 | 0.8 |  |
| **KI67** | **< 20** | 10 | 6.7 | 8 | 6.1 | 1,000 |
|  | **> 21** | 140 | 93.3 | 123 | 93.9 |  |
| **Histotype** | **ILC** | 5 | 3.3 | 8 | 6.1 | 0,708 |
|  | **Apocrine** | 12 | 8.0 | 8 | 6.1 |  |
|  | **Mucinous** | 5 | 3.3 | 3 | 2.3 |  |
|  | **Medullary** | 10 | 6.7 | 6 | 4.6 |  |
|  | **Papillary** | 3 | 2.0 | 0 | 0.0 |  |
|  | **Micropapillary** | 2 | 1.3 | 2 | 1.5 |  |
|  | **Metaplastic squamous** | 3 | 2.0 | 1 | 0.8 |  |
|  | **NST** | 107 | 71.3 | 100 | 76.3 |  |
|  | **Metaplastic** | 3 | 2.0 | 3 | 2.3 |  |
| **TILs** | **Low** | 62 | 55.4 | 51 | 56.0 | 0,733 |
|  | **Moderate** | 42 | 37.5 | 31 | 34.1 |  |
|  | **High** | 8 | 7.1 | 9 | 9.9 |  |
| **AR** | **<1** | 89 | 63.1 | 77 | 60.6 | 0,675 |
|  | **≥1** | 52 | 36.9 | 50 | 39.4 |  |
| **CK5/6** | **<1** | 79 | 57.7 | 74 | 63.2 | 0,365 |
|  | **≥1** | 58 | 42.3 | 43 | 36.8 |  |
| **p63** | **<1** | 122 | 87.8 | 103 | 88.8 | 0,278 |
|  | **≥1** | 17 | 12.2 | 13 | 11.2 |  |
| **IL3** | **No** | 20 | 23.5 | 26 | 31.0 | 0,278 |
|  | **Yes** | 65 | 76.5 | 58 | 69.0 |  |
| **Treatment** | | | | | | |
| **NACT** | **No** | 131 | 87.3 | 94 | 71.8 | 0,001 |
|  | **Yes** | 19 | 12.7 | 37 | 28.2 |  |
| **Anthracycline** | **No** | 62 | 60.2 | 56 | 52.8 | 0,283 |
|  | **Yes** | 41 | 39.8 | 50 | 47.2 |  |
| **Taxane** | **No** | 17 | 16.5 | 24 | 22.6 | 0,264 |
|  | **Yes** | 86 | 83.5 | 82 | 77.4 |  |
| **Antimetabolites** | **No** | 89 | 86.4 | 78 | 73.6 | 0,021 |
|  | **Yes** | 14 | 13.6 | 28 | 26.4 |  |
| **Alkylating agents** | **No** | 74 | 71.8 | 71 | 67.0 | 0,446 |
|  | **Yes** | 29 | 28.2 | 35 | 33.0 |  |

**Table S2. Clinical-pathological features compared with Progression-Free Survival.**

| **Variable/Description** | | **OS** | | | |  |
| --- | --- | --- | --- | --- | --- | --- |
|  |  | **No** | | **Yes** | |  |
|  |  | **n** | **%** | **n** | **%** | ***p* value** |
| **Age (Years)** | **< 50** | 22 | 10.5 | 7 | 9.0 | 0.761 |
|  | **> 51** | 186 | 89.4 | 68 | 90.6 |  |
| **Grade** | **Grade II** | 28 | 13.6 | 9 | 12.5 | 0,814 |
|  | **Grade III** | 178 | 86.4 | 63 | 87.5 |  |
| **pT** | **pT0** | 2 | 1.0 | 0 | 0.0 | 0,016 |
|  | **pT1** | 111 | 53.1 | 19 | 26.4 |  |
|  | **pT2** | 83 | 39.7 | 42 | 58.3 |  |
|  | **pT3** | 10 | 4.8 | 7 | 9.7 |  |
|  | **pT4** | 3 | 1.4 | 4 | 5.6 |  |
| **pN** | **pNX** | 12 | 5.8 | 5 | 6.9 | 0,086 |
|  | **pN0** | 127 | 61.7 | 32 | 44.4 |  |
|  | **pN1** | 6 | 2.9 | 4 | 5.6 |  |
|  | **pN1a** | 33 | 16.0 | 15 | 20.8 |  |
|  | **pN1b** | 2 | 1.0 | 1 | 1.4 |  |
|  | **pN2** | 12 | 5.8 | 8 | 11.1 |  |
|  | **pN3** | 1 | 0.5 | 1 | 1.4 |  |
|  | **pN3a** | 12 | 5.8 | 6 | 8.3 |  |
|  | **pN3c** | 1 | 0.5 | 0 | 0.0 |  |
| **KI67** | **< 20** | 14 | 6.7 | 4 | 5.4 | 1,000 |
|  | **> 21** | 195 | 93.3 | 70 | 94.6 |  |
| **Histotype** | **ILC** | 12 | 5.7 | 1 | 1.4 | 0,231 |
|  | **Apocrine** | 15 | 7.2 | 5 | 6.8 |  |
|  | **Mucinous** | 3 | 1.4 | 5 | 6.8 |  |
|  | **Medullary** | 14 | 6.7 | 2 | 2.7 |  |
|  | **Papillary** | 3 | 1.4 | 0 | 0.0 |  |
|  | **Micropapillary** | 3 | 1.4 | 1 | 1.4 |  |
|  | **Metaplastic squamous** | 3 | 1.4 | 1 | 1.4 |  |
|  | **NST** | 152 | 72.7 | 57 | 77.0 |  |
|  | **Metaplastic** | 4 | 1.9 | 2 | 2.7 |  |
| **TILs** | **Low** | 73 | 51.0 | 42 | 67.7 | 0,086 |
|  | **Moderate** | 57 | 39.9 | 16 | 25.8 |  |
|  | **High** | 13 | 9.1 | 4 | 6.5 |  |
| **AR** | **<1** | 127 | 64.1 | 41 | 56.9 | 0,281 |
|  | **≥1** | 71 | 35.9 | 31 | 43.1 |  |
| **CK5/6** | **<1** | 113 | 58.9 | 42 | 65.6 | 0,337 |
|  | **≥1** | 79 | 41.1 | 22 | 34.4 |  |
| **p63** | **<1** | 169 | 90.4 | 58 | 82.9 | 0,095 |
|  | **≥1** | 18 | 9.6 | 12 | 17.1 |  |
| **IL3** | **No** | 39 | 30.7 | 7 | 16.7 | 0,076 |
|  | **Yes** | 88 | 69.3 | 35 | 83.3 |  |
| **Treatment** | | | | | | |
| **NACT** | **No** | 168 | 80.4 | 58 | 78.4 | 0,712 |
|  | **Yes** | 41 | 19.6 | 16 | 21.6 |  |
| **Anthracycline** | **No** | 88 | 54.3 | 30 | 63.8 | 0,247 |
|  | **Yes** | 74 | 45.7 | 17 | 36.2 |  |
| **Taxane** | **No** | 28 | 17.3 | 13 | 27.7 | 0,115 |
|  | **Yes** | 134 | 82.7 | 34 | 72.3 |  |
| **Antimetabolites** | **No** | 134 | 82.7 | 33 | 70.2 | 0,060 |
|  | **Yes** | 28 | 17.3 | 14 | 29.8 |  |
| **Alkylating agents** | **No** | 115 | 71.0 | 30 | 63.8 | 0,349 |
|  | **Yes** | 47 | 29.0 | 17 | 36.2 |  |

**Table S3. Clinical-pathological characteristics compared with Overall Survival.**

| **Variable/Description** | | **PFS** | | | |  |
| --- | --- | --- | --- | --- | --- | --- |
|  |  | **No** | | **Yes** | |  |
|  |  | **n** | **%** | **n** | **%** | ***p* value** |
| **Age (Years)** | **< 50** | 11 | 9,0 | 18 | 11.11 | 0 .579 |
|  | **> 51** | 110 | 90.91 | 144 | 88.89 |  |
| **Grade** | **Grade II** | 16 | 13 .6 | 21 | 13 .1 | 0 .916 |
|  | **Grade III** | 102 | 86 .4 | 139 | 86 .9 |  |
| **pT** | **pT0** | 2 | 1 .7 | 0 | 0 | 0.002 |
|  | **pT1** | 72 | 60,0 | 57 | 35 .6 |  |
|  | **pT2** | 42 | 35,0 | 83 | 51 .9 |  |
|  | **pT3** | 4 | 3 .3 | 13 | 8 .1 |  |
|  | **pT4** | 0 | 0,0 | 7 | 4 .4 |  |
| **pN** | **pNX** | 8 | 6 .8 | 9 | 5 .7 | 0.000 |
|  | **pN0** | 105 | 89,0 | 53 | 33 .3 |  |
|  | **pN1** | 1 | 0 .8 | 9 | 5 .7 |  |
|  | **pN1a** | 4 | 3 .4 | 44 | 27 .7 |  |
|  | **pN1b** | 0 | 0,0 | 3 | 1 .9 |  |
|  | **pN2** | 0 | 0,0 | 20 | 12 .6 |  |
|  | **pN3** | 0 | 0,0 | 2 | 1 .3 |  |
|  | **pN3a** | 0 | 0,0 | 18 | 11 .3 |  |
|  | **pN3c** | 0 | 0,0 | 1 | 0 .6 |  |
| **KI67** | **< 20** | 9 | 7 .5 | 9 | 5 .6 | 0.509 |
|  | **> 21** | 111 | 92 .5 | 153 | 94 .4 |  |
| **Histotype** | **ILC** | 5 | 4 .2 | 8 | 4 .9 | 0.939 |
|  | **Apocrine** | 9 | 7 .5 | 11 | 6 .8 |  |
|  | **Mucinous** | 3 | 2 .5 | 5 | 3 .1 |  |
|  | **Medullary** | 9 | 7 .5 | 7 | 4 .3 |  |
|  | **Papillary** | 3 | 2 .5 | 0 | 0 |  |
|  | **Micropapillary** | 2 | 1 .7 | 2 | 1 .2 |  |
|  | **Metaplastic squamous** | 3 | 2 .5 | 1 | 0 .6 |  |
|  | **NST** | 84 | 70 | 124 | 76 .5 |  |
|  | **Metaplastic** | 2 | 1 .7 | 4 | 2 .5 |  |
| **TILs** | **Low** | 41 | 48 .8 | 73 | 60 .8 | 0.950 |
|  | **Moderate** | 36 | 42 .9 | 37 | 30 .8 |  |
|  | **High** | 7 | 8 .3 | 10 | 8 .3 |  |
| **AR** | **<1** | 74 | 65 .5 | 93 | 59 .6 | 0 .327 |
|  | **≥1** | 39 | 34 .5 | 63 | 40 .4 |  |
| **CK5/6** | **<1** | 62 | 54 .9 | 92 | 64 .8 | 0 .108 |
|  | **≥1** | 51 | 45 .1 | 50 | 35 .2 |  |
| **p63** | **<1** | 100 | 90 .1 | 126 | 86 .9 | 0 .431 |
|  | **≥1** | 11 | 9 .9 | 19 | 13 .1 |  |
| **IL3** | **No** | 17 | 25,0 | 29 | 28 .7 | 0 .595 |
|  | **Yes** | 51 | 75,0 | 72 | 71 .3 |  |
| **Treatment** | | | | | | |
| **NACT** | **No** | 103 | 85 .8 | 122 | 75 .3 | 0.030 |
|  | **Yes** | 17 | 14 .2 | 40 | 24 .7 |  |
| **Anthracycline** | **No** | 53 | 59 .6 | 65 | 54 .2 | 0.438 |
|  | **Yes** | 36 | 40 .4 | 55 | 45 .8 |  |
| **Taxane** | **No** | 13 | 14 .6 | 28 | 23 .3 | 0.116 |
|  | **Yes** | 76 | 85 .4 | 92 | 76 .7 |  |
| **antimetabolites** | **No** | 80 | 89 .9 | 87 | 72 .5 | 0.002 |
|  | **Yes** | 9 | 10 .1 | 33 | 27 .5 |  |
| **Alkylating agents** | **No** | 65 | 73,0 | 80 | 66 .7 | 0.323 |
|  | **Yes** | 24 | 27,0 | 40 | 33 .3 |  |

**Supplementary Figure 1.** External validation of FOXA1 prognostic value using KM Plotter. Kaplan-Meier curves for (A) distant metastasis-free survival (DMFS), (B) overall survival (OS), and (C) relapse-free survival (RFS) according to FOXA1 mRNA expression (high vs. low, median cutoff) in TNBC/ER-negative patients. Hazard ratios (HR) with 95% confidence intervals and log-rank p-values are shown.

**Supplementary Figure S2.** Predictive performance of FOXA1 mRNA expression for chemotherapy response in TNBC/ER-negative patients using ROC Plotter. Receiver operating characteristic (ROC) curve evaluating the predictive performance of FOXA1 for pathological response. The area under the curve (AUC) was 0.615 (p = 0.00444), with an optimal cutoff of 111, corresponding to a true positive rate (TPR) of 0.66 and a true negative rate (TNR) of 0.55.
